# Experimental H-type and L-type bovine spongiform encephalopathy in cattle: observation of two clinical syndromes and diagnostic challenges

**DOI:** 10.1186/1746-6148-8-22

**Published:** 2012-03-08

**Authors:** Timm Konold, Gemma E Bone, Derek Clifford, Melanie J Chaplin, Saira Cawthraw, Michael J Stack, Marion M Simmons

**Affiliations:** 1 TSE Department, Animal Health and Veterinary Laboratories Agency Weybridge, New Haw, Addlestone, UK; 2 Animal Services Unit, Specialist Scientific Support Department, Animal Health and Veterinary Laboratories Agency Weybridge, New Haw, Addlestone, UK; 3 Central Sequencing Unit, Specialist Scientific Support Department, Animal Health and Veterinary Laboratories Agency Weybridge, New Haw, Addlestone, UK

## Abstract

**Background:**

The majority of atypical bovine spongiform encephalopathy (BSE) cases so far identified worldwide have been detected by active surveillance. Consequently the volume and quality of material available for detailed characterisation is very limiting. Here we report on a small transmission study of both atypical forms, H- and L-type BSE, in cattle to provide tissue for test evaluation and research, and to generate clinical, molecular and pathological data in a standardised way to enable more robust comparison of the two variants with particular reference to those aspects most relevant to case ascertainment and confirmatory diagnosis within existing regulated surveillance programmes.

**Results:**

Two groups of four cattle, intracerebrally inoculated with L-type or H-type BSE, all presented with a nervous disease form with some similarities to classical BSE, which progressed to a more dull form in one animal from each group. Difficulty rising was a consistent feature of both disease forms and not seen in two BSE-free, non-inoculated cattle that served as controls. The pathology and molecular characteristics were distinct from classical BSE, and broadly consistent with published data, but with some variation in the pathological characteristics. Both atypical BSE types were readily detectable as BSE by current confirmatory methods using the medulla brain region at the obex, but making a clear diagnostic distinction between the forms was not consistently straightforward in this brain region. Cerebellum proved a more reliable sample for discrimination when using immunohistochemistry.

**Conclusions:**

The prominent feature of difficulty rising in atypical BSE cases may explain the detection of naturally occurring cases in emergency slaughter cattle and fallen stock. Current confirmatory diagnostic methods are effective for the detection of such atypical cases, but consistently and correctly identifying the variant forms may require modifications to the sampling regimes and methods that are currently in use.

## Background

After bovine spongiform encephalopathy (BSE) was first described as a novel neurological disease in cattle in the United Kingdom (UK) more than 20 years ago [1], subsequent research indicated that it was caused by a single agent based on the clinical [2], pathological [3], molecular [4,5] and biological [6] phenotype. Experimental inoculation of cattle with agents found in transmissible spongiform encephalopathies (TSEs) of other species, such as scrapie [7-9] or chronic wasting disease [10], produced a neurological disease, but in each case the disease was different phenotypically from BSE. The introduction of active surveillance in various countries has resulted in the detection of other, putatively sporadic, forms of BSE in cattle which were characterised by different neuropathological [bovine amyloidotic spongiform encephalopathy (BASE)] [11] or molecular features (L-type and H-type BSE) [12-16] and affected mainly cattle that were 8 years of age or older. Based on the prion protein fragments visualised on a Western immunoblot after proteinase digestion of brain tissue from affected animals, the molecular mass was either lower (L-type BSE, also known as BASE) or higher (H-type BSE) than in the classical form of BSE (C-type BSE). Both forms of atypical BSE were subsequently also identified in the United Kingdom (UK) [17,18]. To date, none of these 'atypical' BSE cases diagnosed in various countries in cattle (*Bos taurus*) have been reported as clinical suspects, suggesting that the diseases are different from classical BSE. Indeed, transmission studies in cattle produced a disease characterised by signs of dullness and difficulty in rising that maintained its molecular phenotype difference to C-type BSE [19,20].

Most of the cases that have so far been identified worldwide [17] were detected through active surveillance programmes. Consequently the volume and quality of material available for detailed characterisation is very limiting due to only medulla at the obex being collected for statutory diagnosis or, particularly in the case of fallen stock, the tissue is badly autolysed.

With the exception of the first reported L-type BSE case [11] there are no field cases in which combined biochemical and pathological findings throughout the brain or peripheral tissues as well as clinical data have been described, to provide a full phenotypic picture of either H or L variants.

The transmissibility of these variant forms has been previously established in experimental models, both in rodents [21-25] where the phenotype was not consistently stable, and in the natural host [19,20,26,27], in different laboratories.

Here, we report on a small transmission study in cattle by the intracerebral route, which was initiated in the UK as part of the activities of the European Union (EU) reference laboratory, to provide tissue for test evaluation and research, and to generate clinical, molecular and pathological data in a standardised way to enable more robust comparison of the two variants. This paper informs on the clinical, pathological and molecular characteristics of L-type and H-type BSE with particular reference to those aspects most relevant to case ascertainment and confirmatory diagnostic sensitivity and specificity within existing regulated surveillance programmes.

## Results

### Animal data and clinical disease

**Figure 1:**
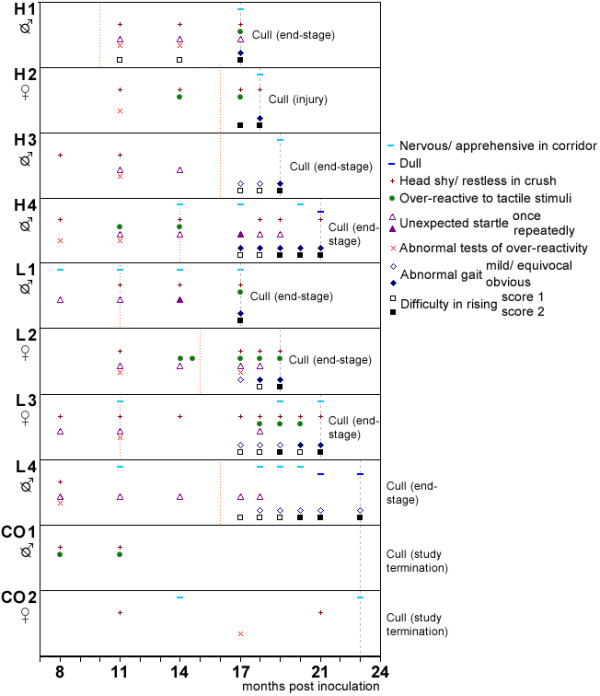
**Selected clinical signs observed over time in L- and H-type BSE cases compared to controls**. Signs are derived from observations and examinations. The horizontal axis represents the time points when neurological examinations were carried out; a symbol at a particular time point indicates that the sign was observed between this and the previous time point. The orange dotted lines indicate the time of estimated clinical onset, the light brown interrupted lines the time of death. Head shy/restless in crush: e.g. head tossing or backing off when faced in crush. Over-reactive to tactile stimuli: e.g. head tossing in response to touching of the head. Abnormal tests of over-reactivity: startle at flash, clipboard test or hand clap, kicking on stick test. Abnormal gait: e.g. dysmetria.

All cattle (H1-4, inoculated with H-type BSE; L1-4, inoculated with L-type BSE and CO1-2, non-inoculated controls) were homozygous for six octapeptide repeats (6:6) and carried silent single nucleotide sequence polymorphisms at codon 78 [L2, H1, H3-4 (heterozygous), CO2 (homozygous)], codon 113 [L1, L3-4, H1-2, CO1 (heterozygous)] or codon 192 [L2 (heterozygous)]. Times of clinical onset and death are indicated in Figure 1.

The clinical presentation appeared to be similar in both H- and L-type BSE-affected cattle. Selected signs of changes in behaviour and mental status, sensation and locomotion observed over time are displayed in Figure 1, which also shows the findings in controls for comparison. The estimated clinical onset was based on the presence of difficulty in rising (H2), in combination with regular unexpected startle (H1 and L4) or with suspected gait abnormality (H3), or the presence of behavioural and sensory changes (L1-3 and H4).

Animal H2 injured itself when caught between hurdles in the pen, which resulted in culling at an earlier clinical stage compared to the others.

#### Changes in behaviour, activity, mental status and sensation

All inoculated cattle displayed signs of a 'nervous disease form', characterised by over-reactivity to external stimuli, apprehension or anxiety (see Additional file 1: 'H-type BSE (H1)' and Additional file 2: 'L-type BSE (L1)' showing over-reactivity and apprehension during the examination). First noticeable from 8-11 months post inoculation (mpi) were restlessness and head shyness when the cattle were approached from the front whilst restrained in a crush, characterised by sudden backing off, head shaking or head tossing. Contrary to the control cattle that also displayed this behaviour around that time, the BSE-infected cattle continued to display this sign on subsequent examinations, which was also accompanied by sensory changes, such as increased over-reactivity to tactile facial stimuli (head tossing, nose wrinkling, snorting) or visual stimuli (jerking, tossing or shaking of the head) during assessment of cranial nerve function [compare signs 'head shyness' and 'over-reactivity to tactile stimuli' between H/L-type BSE cases and controls in Figure 1; see also Additional file 3: 'Control (CO1)', which shows the lack of over-reactivity in this control animal prior to cull]. There was also a noticeable change in the behaviour when the animals were released in the corridor: they became more reluctant to move in the corridor, stopping at grooves on the floor [see Additional file 1: 'H-type BSE (H1)' of this steer stopping at grooves on the floor], whereas they had previously been curious and active in the corridor like the control cattle [see Additional file 3: 'control (CO1)' showing this control animal running along the corridor and stopping to sniff objects]. Behavioural or sensory changes also included the increased occurrence of startle reactions, spontaneously or in response to sudden external stimuli, which were not observed in control cattle [compare the occurrence of 'unexpected startle' between H/L-type BSE cases and controls in Figure 1; see also Additional file 4: 'H-type BSE (H4a)' showing a steer with startle compared to a control in similar circumstances]. Repeated startle in response to tests of over-reactivity (e.g. flash test, clipboard test) was however not or not consistently observed, similar to control cattle (see Figure 1). Forceful kicking in response to touching of the hind limbs with a flexible stick was only observed in an L-type BSE-inoculated heifer (L2).

One H-type BSE-inoculated steer (H3) had a 'panic attack' at 19 mpi: it panicked during cleaning of the pen, ran into the hay rack and the wall of the pen, slipped after circling in the pen and fell to the floor in lateral recumbency with its legs thrashing for approximately 90 seconds. It subsequently remained still in the same position for approximately 5 minutes before righting itself. The steer eventually got up 3 minutes later rising with its forelimbs first.

Two cattle (H1 and L2) developed marked distress to restraining of the head, characterised by open-mouthed roaring prior to cull.

Having initially displayed the 'nervous form' the last remaining animal in each group (L4 and H4) later developed a more 'dull disease form' from 21 mpi, where the initial over-reactivity to tactile stimuli disappeared and the animal appeared less interested in its surroundings, sometimes standing in the pen with its head lowered [see Additional file 5: 'H-type BSE (H4b)', which shows this animal standing with low head carriage and little over-reactivity to tactile stimuli].

#### Changes in gait and coordination

All atypical BSE-infected cattle developed difficulty in rising, which usually coincided with the development of gait deficits (see Figure 1), predominantly hind limb hypermetria [see Additional files 1: 'H-type BSE (H1)', 2:' L-type BSE (L1)' and 5: 'H-type BSE (H4b)' showing all animals with difficulty in rising (score 2) prior to cull; hind limb hypermetria is also evident in L1]. Difficulty in rising was not always consistently displayed after it was first observed; there could be a period of up to five weeks of normal rising behaviour when weekly camera observations were analysed, although it became more regular when cattle displayed score 2 (obvious difficulty in getting up). Heifer L3 was found in lateral recumbency, unable to right itself at 21 mpi, although it managed to get up when placed into sternal position.

Altered lying down behaviour was observed in all H-type BSE-inoculated cattle and one L-type BSE-inoculated steer (L4) at some time during the incubation period (usually only once and never at the last camera observation prior to cull) scoring 3 in three and 2 in two (H4 and L4) cattle. It was also observed once in the control steer (score 3).

Tremor was not displayed in any of the cattle throughout the course of the disease. Loss of weight or bodily condition was not a consistent feature and only observed prior to cull in one L-type (L1) and two H-type BSE affected cattle (H1-2).

### Haematology and blood biochemistry

Serum aspartate aminotransferase (AST) and creatine kinase (CK) activity were increased in two H-type BSE cases, the injured H2 and H3, which was culled shortly after the thrashing episode during the 'panic attack': AST activity was 492 units/l in H2 and 156 units/l in H3, compared to the reference range of 78-132 units/l. CK activity was 104,133 units/l in H2 and 2,136 units/l in H3, with a reference range of 35-280 units/l. H2 also presented with neutrophilia (14,900/μl; reference range: 600-4,000/μl).

One control and two L-type BSE cases had reduced blood manganese (reference range: 330-350 nmol/l): CO1: 80 nmol/l, L3: 83 nmol/l and L4: 89 nmol/l.

### Postmortem test findings

**Figure 2:**
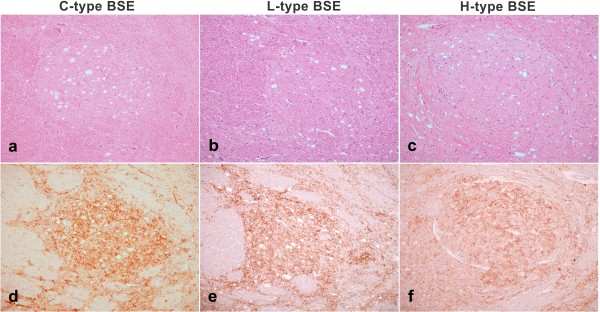
**Vacuolation and PrP^d ^immunolabelling in the solitary tract nucleus of classical and atypical BSE cases**. a-c) Haematoxylin and eosin (H&E) stained sections. d-f) Sections immunolabelled with monoclonal antibody (mAb) R145. Comparison of a C-type BSE case (intracerebrally inoculated case 163/95; a and d) with an L-type BSE case (L3; b and e) and an H-type BSE case (H1; c and f). In all three cases vacuolation is present, and immunolabelling is extensive and presents with particulate neuropil labelling as the predominant form.

Vacuolar lesions consistent with TSE were observed throughout the neuraxis in both the H-type and L-type BSE cases. At the obex, the neuroanatomical distribution of vacuolation, and its appearance, were not distinguishable from C-type BSE (Figure 2). However, the amount of vacuolation, relative to observations in positive control animals challenged with C-type BSE by the same route (data from previous studies [9]), appeared to be increased in more rostral brain areas, noticeably the frontal cortex.

#### Immunohistochemical examination

**Figure 3:**
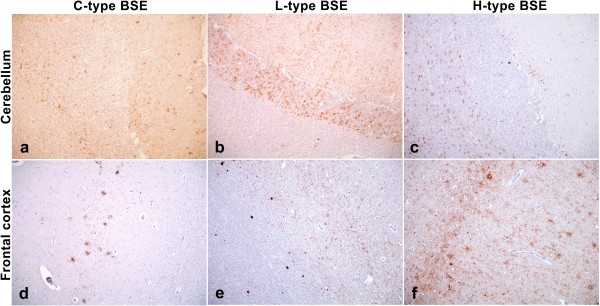
**PrP^d ^immunolabelling in cerebellum and frontal cortex of classical and atypical BSE cases**. Immunolabelling with mAb R145. a-c) Cerebellum of C-type BSE (intracerebrally inoculated case 163/95; a), L-type BSE (L1; b) and H-type BSE (H3; c). e-f) Frontal cortex of C-type BSE (163/95; d), L-type BSE (L3; e), and H-type BSE (H3; f). Of particular interest in the cerebellum is the diffuse and even involvement of both the molecular and granular layers in L-type BSE, while in the cerebellum of the H-type BSE case the most pronounced labelling is in the white matter. In the cortex small aggregated deposits, mostly in the white matter, predominate in the L-type BSE case, while the H-type BSE case resembles the classical BSE case with stellate forms throughout the cortical grey matter.

**Figure 4:**
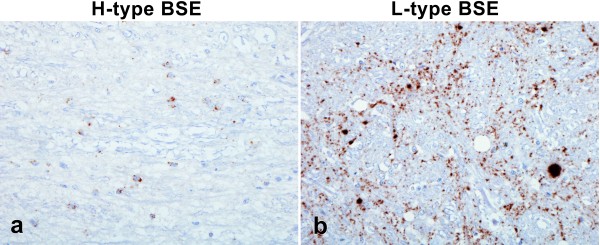
**Differences in PrP^d ^immunolabelling between L-type and H-type BSE**. Immunolabelling with mAb 145, which can be used to distinguish H- and L-type forms of disease at the level of the obex. a) Labelling of the oligodendroglia in the pyramidal tract in an H-type BSE case (H3). b) Aggregated forms within the reticular formation of an L-type BSE case (L3).

Immunolabelling was widespread throughout the neuraxis in both the H- and L-type BSE cases, but with patterns of immunolabelling that were distinct from one another and distinguishable from that seen in C-type BSE [5,28] (Figure 3). As reported previously [11] the L-type cases showed extensive small plaque-like deposits throughout the cortical white matter, with moderate amounts of particulate immunolabelling in the neuropil (Figure 3). Perineuronal labelling was also prominent. Another striking feature of the L-type BSE cases was the immunolabelling in the cerebellum, with a very homogeneous involvement of both the molecular and granular layers, reminiscent of the pattern described for atypical scrapie in sheep. By comparison, H-type BSE cases showed fewer plaque-like deposits, and the grey matter of the cerebral cortex displayed prominent stellate labelling in addition to particulate forms (Figure 3). The labelling in the molecular and granular layers of the cerebellum was minimal and less uniformly distributed. In the H-type BSE cases, the most striking immunohistochemical feature was widespread glial labelling throughout the white matter of the spinal cord (Figure 4) and the cerebellum (Figure 3).

At the obex, the amount and distribution of immunolabelling made it difficult to see obvious differences between these cases and C-type BSE, but close examination revealed phenotype-specific features in this area too, specifically labelling in white matter tracts (Figure 4a) in the H-type BSE cases, and small aggregated forms of immunolabelling (Figure 4b) throughout the reticular formation in L-type BSE cases.

Widespread immunolabelling was seen throughout the dorsal and ventral horns at all levels of the spinal cord as in C-type BSE.

Immunolabelling was also present in the muscle spindles of the extraocular muscles and in the trigeminal ganglion of both types. Where muscle spindles were found in other muscles (triceps in one H-type BSE case, and medial gluteal in one L-type BSE case) these were also immunolabelled.

No immunolabelling was observed in the lymphoid tissues or the enteric nervous system in any of the challenged animals.

#### Western immunoblot examination

**Figure 5:**
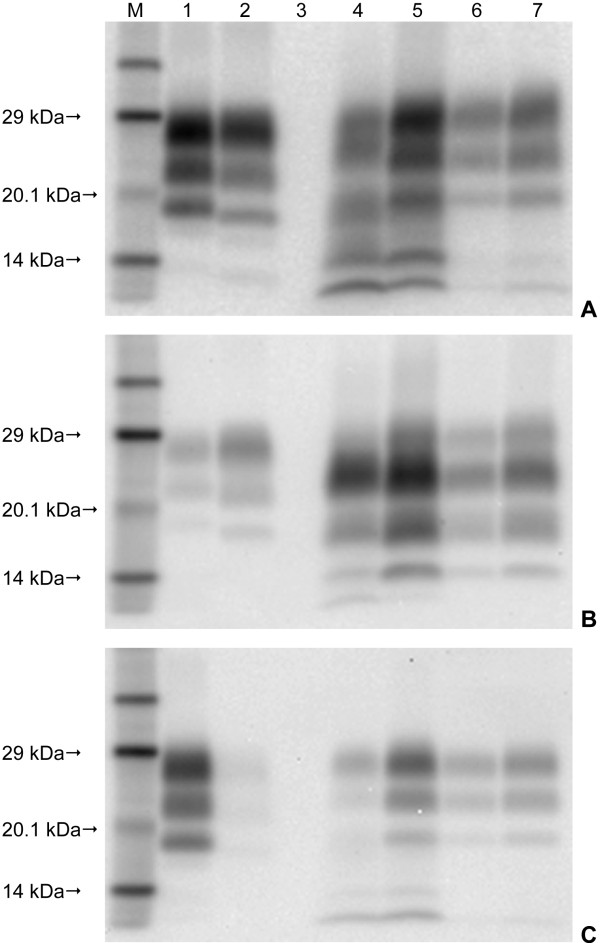
**Western immunoblot of medulla samples from the four H-type BSE recipients using three different antibodies**. A: mAb SHA31, B: mAb SAF84, C: mAb P4. Lane Key: M Molecular mass marker. 1 Classical scrapie control 1:2 dilution. 2 C-type BSE control 1:40 dilution. 3 Blank lane. 4 H1. 5 H2. 6 H3. 7 H4.

**Figure 6:**
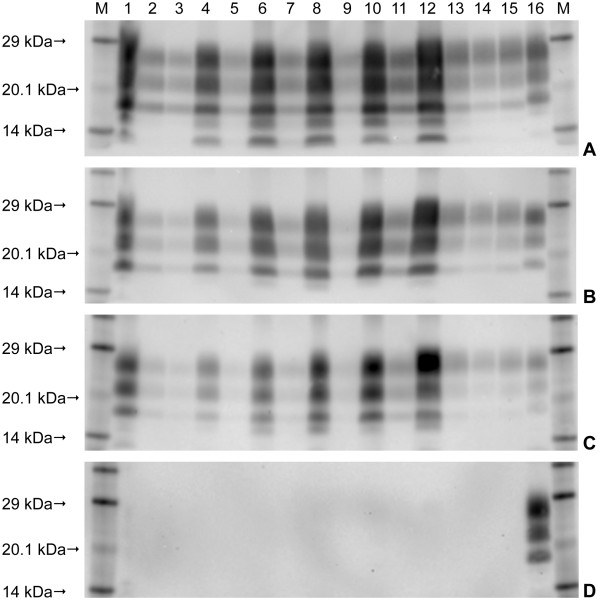
**Western immunoblot of medulla samples from the L-type BSE donor animal and the four recipients using four different antibodies**. A: mAb SHA31, B: mAb 6H4, C: mAb F99, D: mAb P4. Samples were loaded neat and at dilutions indicated in the key. Lane Key: M Molecular mass marker. 1 Donor neat. 2 Donor 1:20. 3 Donor 1:40. 4 L1 neat. 5 L1 1:10. 6 L2 neat. 7 L2 1:10. 8 L3 neat. 9 L3 1:20. 10 L4 neat. 11 L4 1:10. 12 C-type BSE control neat. 13 C-type BSE control 1:20. 14 C-type BSE control 1:40. 15 Standard bovine C-type BSE control 1:40. 16 Standard ovine scrapie control 1:2.

**Figure 7:**
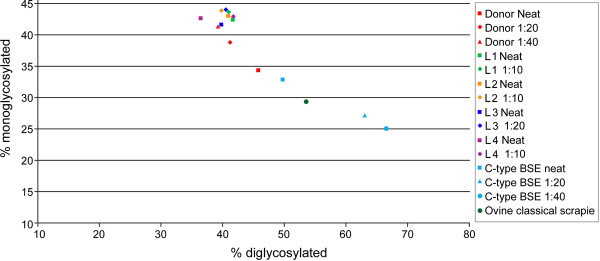
**Glycoform profiles for all four L-type BSE recipients compared to C-type BSE and scrapie using mAb SHA31**. The glycoform profiles in this scattergram are displayed as the percentage signal of the diglycosylated protein band plotted against that of the monoglycosylated band. The percentage of diglycosylated PrP^res ^is reduced in L-type BSE cases compared to C-type BSE or classical scrapie.

Western immunoblot was carried out on fresh medulla tissue from each of the eight challenged animals. The four H-type BSE challenged animals showed the characteristic profile of the proteinase-resistant fragment of the disease-associated prion protein (PrP^res^) associated with this form of disease; a high molecular mass migration of the unglycosylated band with mAb SHA31, a similar intensity of signal with mAb P4, and a downward shift of the PrP^res ^bands with an additional low molecular mass band observed with mAb SAF84 (Figure 5). The four L-type BSE challenged animals were less easily distinguished as the migration of the unglycosylated PrP^res ^band was similar to the classical BSE control when detected with mAbs SHA31 and SAF84, and they were also not detected with mAb P4. L-type BSE is also expected to have an increase in the proportion of the monoglycosylated band or a more equal distribution of PrP^res ^between the di and monoglycosylated bands. Although this characteristic was present in all four samples, visually the difference was very subtle, and when the signal intensity of a sample was particularly strong the difference was not readily observed until the sample had been diluted (Figure 6). Glycoform analysis of the relative quantity of the di- versus monoglycosylated bands confirmed the difference between the L-type BSE cases and the C-type BSE control (Figure 7).

**Figure 8:**
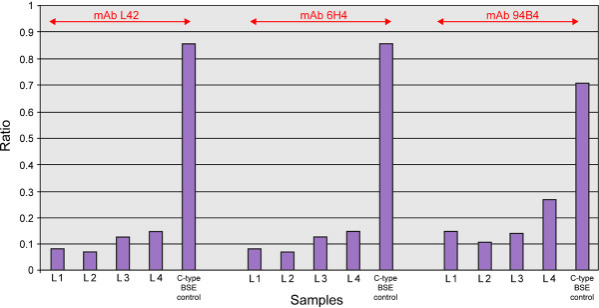
**Bar chart of proteinase K susceptibility signal ratio in L-type recipients compared to C-type BSE using three different antibodies**. The PK susceptibility signal ratio is < 0.6 for all four L-type BSE recipients and > 0.7 for the C-type BSE control sample.

When disease-associated prion protein (PrP^d^) from C- and L-type BSE cases is digested under two proteinase K (PK) conditions, mild and stringent, PrP^res ^from C-type BSE remains intact whereas PrP^d ^from L-BSE is largely destroyed in the stringent preparation. The four L-type BSE samples were digested using stringent PK treatment whereas the classical BSE control samples were not. When the optical density ratios for the immunoblots produced using mild and stringent PK conditions were displayed by a bar chart the C-type BSE controls were above 0.7 whilst the L-type BSE cases were below 0.6, irrespective of which of the three antibodies was used (Figure 8). Overall, the results for these samples conformed to the recognised criteria for L-type BSE [12].

## Discussion

Classical BSE was first described as a novel neurological disease in cattle, which displayed overt clinical signs characterised by changes in behaviour, sensation and locomotion [1], which led to reporting of many clinical cases in the UK [29]. Atypical BSE, by contrast, has so far not been confirmed in clinical suspects: it has been found in either apparently healthy cattle at abattoirs, cattle that were subject to emergency slaughter or in fallen stock and may suggest that the clinical presentation is different to C-type BSE. Indeed, previously published experimental transmission studies of atypical BSE to cattle conducted elsewhere describe H-type and L-type BSE as diseases characterised by dullness, low head carriage, inactivity or lack of nervousness whereas cattle with classical BSE presented with nervousness or anxiety and hypersensitivity to various external stimuli [19,20,26,27]. However, hypersensitivity to facial tactile or other external stimuli was also observed in H- or L-type BSE although the study did not use unchallenged controls for comparison with clinically 'normal' cattle [19,27]. The clinical signs in the study reported here resembled those observed in the German study [27], with no difference in the clinical presentation between H-type and L-type BSE, but separation from other cattle and low head carriage suggestive of dullness were not a consistent finding. In fact, the majority of cattle in the current study showed no signs of dullness, even on closed circuit television observations, but were over-reactive, characterised by over-reactivity to tactile facial and visual stimuli (menace response testing) and displayed unexpected startle responses throughout the clinical phase. Cattle intracerebrally inoculated with classical BSE were not included but previous transmission studies of classical BSE in Holstein-Friesian cattle conducted at the Veterinary Laboratories Agency confirmed that over-reactivity and nervousness were a feature of classical BSE [19,20,30]. There is, however, variation in the severity of behavioural and sensory changes in cattle intracerebrally infected with classical BSE, particularly influenced by the frequent handling of cattle in experimental studies, which may lead to habituation to clinical tests. In the authors' opinion, the 'nervous syndrome' of cattle inoculated with atypical BSE has some resemblance to the clinical presentation of classical BSE in some cattle [30]. Notably different to classical BSE were, however, the absence of tremor in all atypical BSE cases, both H- and L-type, and the presence of difficulty in rising, accompanied by dysmetria, early in the disease. In the experimental cases produced in the Italian study (L-type BSE), difficulty in rising and ataxia were surprisingly rare and not observed, respectively [19], even though the inoculum in the present study was also derived from an Italian BASE case.

We previously employed identical clinical methods to describe ovine scrapie transmitted to cattle, which also presented with a dull (low head carriage and head resting against objects) and nervous form (nervousness and over-reactivity), and all cattle developed difficulty in rising [9]. Different prion strains in cattle appear to produce two main clinical phenotypes (dull and nervous form), with classical BSE being the most uniform and - with respect to over-reactivity - clinically the most extreme phenotype.

None of the cattle inoculated with atypical BSE in the current study progressed to permanent recumbency, which has been reported to occur within few days after the display of evident ataxia [27], even though they all had difficulty in rising. If this is a consistent feature of atypical BSE whereas over-reactivity is present in a milder form than classical BSE, it would explain why case reports of naturally occurring atypical BSE describe this disease in downer cows [14,17,31].

Our previous recommendation to detect classical BSE cases in recumbent casualty slaughter cattle on clinical grounds using the display of repeated startle responses to tests of over-reactivity and abnormal limb position in combination with low heart rate or poor body condition as criteria [32] may not have detected the atypical BSE cases in the present study but they were culled prior to permanent recumbency. None of the affected cattle were bradycardic at clinical end-stage (heart rate not determined for case H-2) as reported separately [33] but unexpected startle was observed. Hence, atypical BSE should be considered as differential diagnosis in any recumbent animal, which - based on naturally occurring cases [17] - is 8 years of age or older and has a history of or presents with over-reactivity to various external stimuli.

The clinical duration of atypical BSE in the current study was 4-10 months for L-type BSE and 2-7 months for H-type BSE, which is considerably longer than reported by others [19,20,27] except for an experimental H-type BSE study in Japan [26]. As the survival times or incubation periods (time from inoculation to cull at clinical end-stage) and pathology were similar, the discrepancy with other studies may be explained by the difficulty in defining the exact clinical onset, which is made retrospectively and based on the presence of various clinical parameters, which may overlap with those seen in 'normal' cattle. For example, the presence of marked over-reactivity to touch, which diminished on subsequent observations, was also seen in control cattle. However, startle responses to unexpected (familiar) stimuli were not seen in controls and hence - when displayed in combination with other signs, e.g. head shyness - were interpreted as sign indicating clinical onset. Similarly, difficulty in rising was only seen in inoculated cattle and thus also used as a marker for clinical onset. On the other hand, it is possible that the differences in the clinical duration were attributable to the particular cattle breeds used in each study. Indeed, a significantly shorter clinical duration (and incubation period) was reported for Holstein-Friesian cattle compared with Alpine brown cattle intracerebrally inoculated with BASE brain [19]. Analysis of the clinical picture of classical BSE using surveillance data has shown that some statistically significant differences in the clinical sign frequency may exist between different herd types (beef and dairy cattle) and different breeds of dairy cattle [29], which may be due to different methods of management or due to the different temperaments of cattle. However, it is difficult to conclude that there are indeed breed-specific differences in the clinical presentation since the display of individual signs can generally be variable, even if a detailed clinical examination is conducted [34], and depend on other factors, such as duration of illness and age at onset [29].

PrP genotyping revealed that all cattle had six octapeptide repeats and no novel polymorphisms were detected. The only mutations detected were 'silent' in that they do not affect the PrP protein sequence. The lack of wild type sequences and the comparatively high number of cattle with the P113 silent mutation (found in only three of 118 Holstein-Friesians examined in the UK [35]) was unusual but may be due to the breed studied; its occurrence was higher in a study of predominantly beef breeds in the United States [36].

The development of the dull syndrome in only those cattle that had the longest clinical duration but previously exhibited the nervous syndrome may suggest that all infected cattle might have eventually developed the dull syndrome. Based on the range of clinical signs displayed by all cattle prior to cull, which was considered to be the clinical end-point, there was however no evidence that the cattle exhibiting the dull syndrome were in a more advanced stage of disease than the cattle exhibiting the nervous syndrome at cull. The display of either the nervous or dull syndrome at end-stage in both inoculation groups did not appear to be influenced by the prion protein genotype: both syndromes were displayed by cattle with the same genotype. Equally, the observed differences did not appear to have been attributable to gender. As the majority of the brain from both cattle with the dull syndrome was frozen, it was not possible to determine whether the pathological phenotype was different; accumulation of the disease-associated prion protein (PrP^d^) at the level of the obex was similar for all cases. The lack of association between clinical presentation and PrP^d ^accumulation in the brain has been previously described for scrapie in cattle [9] and goats [37].

Contrary to the findings reported for BASE/L-type BSE in the Italian study, we did not observe muscle atrophy preceded by fasciculations, which were attributed to a lower motor neuron disease [19]. Serum CK and AST, which are useful in the diagnosis of diseases of the muscle, even though they may not provide information about the origin (myopathy or neuropathy) [38], were only altered in two cattle that had muscular damage due to trauma. The finding of behavioural, sensory and locomotor signs in cattle in the present study is suggestive of a brain disease, with dysmetria likely to be caused by cerebellar dysfunction. The 'panic attack' of one L-type BSE-inoculated steer, which thrashed its legs about whilst in lateral recumbency, could even be interpreted as cerebellar convulsions triggered by sudden disturbance [39]. However, other cerebellar signs, such as an abnormal menace response or intention tremor, were not observed. The dysmetric limb movements were also not considered to be so severe that they would have caused difficulty in rising - cerebellar diseases do not cause loss of muscle strength [40] - although rising with the forelimbs first, which was seen occasionally, is suggestive of some inability to coordinate the movements to stand. The hind limb joints were inspected in five atypical BSE cases and both controls and - with the exception of one H-type BSE case (H1) that presented with mild joint degeneration of both stifles - none presented with abnormalities that would have explained the observed gait abnormalities. PrP^d ^immunolabelling was observed throughout the spinal cord of both L-type and H-type BSE cases, although this also occurs in experimentally infected, classical BSE cases without causing difficulty rising [28]. H-type BSE cases presented with marked PrP^d ^accumulation in glia within the white matter at every level examined but there was no apparent difference in the observed gait changes between L-type and H-type BSE cases.

Elevated blood manganese has been reported for cattle experimentally infected with classical BSE [41]. A similar phenomenon was not observed in the present study where two L-type BSE cases had abnormally low levels of blood manganese, similar to one control.

For the purposes of active screening and confirmatory diagnosis it is reassuring that the obex is involved in both H- and L-type BSE, even after intracerebral inoculation of cattle, but it is concerning that discriminating phenotypically between the different forms is not necessarily straightforward if only the brainstem (obex) is available, despite significant qualitative differences in other areas of the brain. This may in part be due to the large quantities of PrP^d ^which have accumulated as a consequence of this experimental challenge being intracerebral, since immunolabelling in the brainstem of field cases has been reported to be much less, for the L-type in particular [11,42], although it may also be a result of these animals being allowed to progress to clinical end-stage, rather than being killed for other reasons, and possibly detected at an earlier point in disease development.

However, in this study the immunohistochemical labelling of H- and L-type BSE differed substantially from each other and from C-type BSE in other areas of the central nervous system. Greater qualitative discrimination was possible examining more rostral areas of the brain, and particularly the cerebellum, despite this area not necessarily displaying the greatest amount of PrP [43]. This offers the possibility of amending routine surveillance sampling to include cerebellum taken through the foramen magnum, as is currently required for sheep, thereby enhancing the discriminatory potential of the confirmatory testing. The presence of immunolabelling in muscle spindles and the trigeminal ganglion is consistent with C-type BSE. Current study design does not allow ascertainment of whether this happens at an early stage in the disease, or as a centrifugal spread in end-stage disease as is speculated for C-type BSE [28,44,45].

The absence of detectable involvement of the lymphoreticular and enteric nervous system is consistent with many observations for C-type BSE, and other reports of experimental H-BSE [26] although for C-type BSE it has also been demonstrated that infectivity can be detected in the absence of identifiable PrP^d ^in *in vitro *tests [46,47]. Besides, all cattle were challenged intracerebrally, thus by-passing the gastro-intestinal tract and possible uptake of the agent through the gut.

Interestingly, the H-type BSE challenged animals in this study did not develop plaques like the animals in another experimental challenge study conducted in Japan [26], which also displayed shorter incubation periods and a more restricted range of clinical signs. Unlike the Japanese study, we only used one antibody - the rat monoclonal R145 which recognises the C-terminal of PrP. However, the range of antibodies used in the Japanese study all demonstrated strongly immunolabelled plaque formations, despite more variation in intracellular labelling, so it would appear to be a specific morphological difference between the animals in these studies.

The very small number of animals represented in these studies means that it can only be speculated whether such changes reflect a degree of natural variation in the donor animals, or whether there are as yet unrecognised influences from recipient host factors.

When Western immunoblotting is used for diagnostic or confirmatory purposes, H-type BSE can readily be identified by its characteristic profile even when the signal is strong. In contrast, the characteristics of L-type BSE appear to be more subtle and the results here suggest that when the signal is too intense it is not readily differentiated by visual examination from a classical BSE profile. This observation has led to the routine dilution of samples from any positive BSE case that is subjected to Western immunoblotting under UK surveillance in order to maximise the opportunity for initial identification of this form of atypical BSE and to allow such samples to be subjected to further investigation for full characterisation.

## Conclusions

Cattle experimentally infected by intracerebral inoculation with L-type or H-type BSE present with two clinical phenotypes, either dull or nervous forms, which may be less clinically overt than classical BSE although difficulty in rising is consistently displayed. This may explain the detection of naturally occurring cases in apparently healthy or emergency slaughter cattle and fallen stock. Current screening and confirmatory diagnostic methods are effective for the detection of such atypical cases, but consistently and correctly identifying and discriminating the variant forms may require modifications to the sampling regimes and methods that are currently in use.

## Methods

All procedures involving animals were approved by the Home Office of the UK government according to the Animal (Scientific Procedures) Act 1986.

### Inocula

Brain homogenate (in 10% w/v solution in sterile saline) was prepared from a French cow with H-type BSE (code: ESB-H-07-0644) and an Italian cow with L-type BSE (code: 141387/02). Prior to inoculation, the inocula were either heat treated (twice for 15 minutes at 70°C; H-type BSE) or treated with antibiotics (L-type BSE) according to established methods [48].

### Animals

Ten calves were born from heifers imported from Denmark (Danish Holstein, Danish milking red) that were crossed with Aberdeen Angus. EDTA blood was collected from each animal prior to inoculation for DNA extraction to determine the PrP gene open reading frame polymorphisms according to published methods [35]. Groups of four calves were intracerebrally inoculated with 1.0 ml of either L-type or H-type BSE brain homogenate at 10-11 months of age; the remaining two cattle were not inoculated and served as controls (for inoculation procedure please refer to [48]).

### Husbandry and routine procedures

Both inoculated groups were housed in medium security accommodation with no contact to each other, with separate pen entrances and separate equipment. Control cattle were initially housed on pasture and later moved to the medium security accommodation to serve as companions of the last remaining inoculated steer in its group. All housed cattle were given hay *ad libitum *and a daily concentrate ration free from meat and bone meal. Pens were cleaned out daily by husbandry staff. Routine procedures included quarterly weighing of housed cattle and regular blood sampling for archiving purposes [quarterly up to 16 mpi, monthly thereafter].

### Clinical assessments

All animals were clinically examined prior to inoculation to confirm their suitability for the study.

#### Observations by husbandry staff

Animals were checked daily by animal husbandry staff and any unusual behaviour during feeding or cleaning of the pens was recorded in a daybook. This was extended to include assessment of the rising behaviour of all housed cattle from 17 mpi when difficulty in getting up was first noticed. A scoring system for the rising behaviour was used:

score 0 = getting up without delay, rising on hind limbs first;

score 1 = slight difficulties getting up, e.g. the phase of rising on the hind limbs seems to be delayed;

score 2 = obvious difficulties getting up with rising on the fore limbs first or dragging the body along the floor before getting up;

score 3 = unable to get up, the animal may attempt to do so but does not succeed.

#### Neurological examinations

Neurological examinations were conducted quarterly from 8 mpi and monthly from 17 mpi when the first animal was culled at clinical end-point. Clinical examinations consisted of a neurological examination according to a standard protocol [48], which included inspection of the retina and tests of over-reactivity, such as testing the response to camera flash ('flash test'), to hand clapping and a metallic bang, to a clipboard waved in front of the animal ('clipboard test') and to touching of the hind limbs with a flexible stick ('stick test') [49].

#### Behavioural observations

Behavioural observations for 15 minutes per pen to assess the animals' behaviour and mental status and reactivity [48] were carried out weekly between 11 a.m. and 12 noon from 9 mpi. Observations of the two controls commenced from 19 mpi when they were moved from the pasture. Observations were conducted without interaction of the observer except for the 'hand approach test' (moving the hand towards the animal when close to the observer at the door) or the clipboard test at the end of the observation period if an animal did not approach.

#### Observations by closed circuit television

The two pens housing the inoculated cattle were monitored during daytime by two cameras each (AXIS 209FD with Camera Station software version 3, AXIS Communications, Lund, Sweden) mounted in opposite corners. Weekly camera observations were conducted from 16 mpi (for controls three months later) and comprised the study of selected animals' behaviour and activity associated with BSE [50] for 15 minutes between 10 a.m. and 12 noon (not on the same day as the behavioural observations) as well as a scan of the same day to assess the rising and lying down behaviour of each animal. The scoring system for rising behaviour was identical to that used by animal husbandry staff. Lying down behaviour was scored as follows:

score 0 = lying down without delay or difficulty;

score 1 = trying to lie down but incomplete/gets up again;

score 2 = goes down on knees then slow to lower hindquarters.

### Clinical end-point

Cattle were culled by intravenous injection of pentobarbitone when they reached clinical end-point, which was documented in the Home Office project licence. Based on previous experience with classical BSE, the end-point was reached when cattle displayed signs in at least two of the three categories 'changes in mental status, behaviour and activity', 'changes in sensation' and 'changes in posture and movement'. Euthanasia would also be required if cattle displayed severe weakness likely to cause recumbency, difficulty in eating or drinking or any behaviour indicating pain or any other significant distress. Control cattle were culled when the last inoculated animal was culled.

### Postmortem diagnostic tests

#### Clinical chemistry and haematology

Blood was collected from each animal prior to cull for routine haematological (including differential cell count) and blood biochemical examination (liver enzymes and CK, total protein, albumin and globulin, urea, creatinine, bilirubin, β-hydroxybutyrate, selected electrolytes and minerals, vitamin E, glutathione peroxidase and fibrinogen and haptoglobin). Deviations from 'normal' values were based on published reference ranges [51].

#### Histopathology and immunohistochemistry

The brain was removed and a piece of the medulla at the level of the obex fixed in 10% formal saline for histopathological examination. The remainder of the brain was either frozen at -80°C (second and fourth animal culled in each group) or half fixed and half frozen (first and third culled animal and controls). Sections representing standardised levels of the fixed brain [3,5] were prepared from paraffin wax-embedded blocks using routine histological methods and stained with H&E for the detection of vacuolar changes as described previously [3]. Data generated from animals experimentally inoculated with C-type BSE by the intracerebral route in a previous study [52,53] were used for comparison. This same data was used previously as a comparator for experimental scrapie in cattle [9].

Further sections were immunolabelled with the rat anti-PrP mAb R145 (Animal Health and Veterinary Laboratories Agency, Addlestone, UK) for the detection of PrP^d ^as described in detail elsewhere [5,28].

Immunohistochemical examination was also performed on spinal cord sampled at four levels (C_6-7_, T_4-5_, T_9-10 _and L_2-3_), mesenteric lymph node, distal ileum, palatine tonsil, medial retropharyngeal lymph node, trigeminal ganglion and extraocular muscles, together with samples from the triceps, medial gluteal and semitendinosus muscles.

#### Western immunoblotting

Fresh, frozen samples of caudal medulla were processed using the BioRad TeSeE universal Western blot kit (BioRad Laboratories, Marnes-La-Coquette, France) following the manufacturers instructions. Essentially, the samples were processed, electrophoresed, transferred and detected with the following panel of mAbs. For H-type samples; SHA31 targeting the PrP amino acid (aa) residues 156-163 (BioRad Laboratories, included in the kit), P4 (aa 97-112; R-BioPharm, Darmstadt, Germany) and SAF84 (aa173-178; Cayman Chemicals, Ann Arbor, USA). For L-type BSE samples; SHA31, P4, 6H4 (aa 155-163; Prionics, Schlieren-Zurich, Switzerland) and F99 (aa 228-233; VMRD, Pullman, USA). Where necessary, sample dilutions were carried out in Laemmli sample buffer (BioRad Laboratories) prior to loading on the gel. The signal was visualised by chemoluminescence and the image captured using a Fluor-S multiImager (BioRad Laboratories). The 20% homogenate preparations from the L-type BSE cases were also subjected to mild and stringent PK digestion conditions: stringent digestion was 500 μg/ml PK at pH 8.0, and mild digestion was 50 μg/ml PK at pH 6.5 prior to immunoblotting, as described [12], and detected using mAbs L42 (aa 153-171; R-BioPharm), 6H4 and 94B4 (aa 198-205; donated by Jan Langeveld, CVI Lelystad, Netherlands).

Quantity One software (BioRad Laboratories) was used for analysis of the glycoprofiles and PK susceptibility ratio, in accordance with the guidelines provided by the TSE EU Reference Laboratory [54].

## Competing interests

The authors declare that they have no competing interests.

## Authors' contributions

Clinical examinations and weekly observations were carried out by TK, supported by GEB. GEB analysed the camera observations. DC performed the inoculations and provided clinical support. MJC and MJS were responsible for the Western immunoblotting and SC was responsible for the genotyping. Neuropathological examinations were conducted by MMS who also managed the project. TK and MMS drafted the manuscript with contributions by MJC, MJS and SC. All authors read and approved the final manuscript.

## Supplementary Material

Additional file 1 **H-type BSE (H1) H-type BSE-inoculated steer at 17 mpi (prior to cull), 'nervous form'**. This steer displays head shyness in the crush (over-reactivity when approached from the front) and tosses its head in response to touching of the head with artery forceps. It displays mildly apprehensive behaviour, characterised by stopping at grooves or avoiding the drain on the floor (note also the mild startle with twitching of the forelimbs after the animal vocalises in the corridor and trots towards the camera). There is mild hind limb hypermetria. It has considerable difficulty in rising, dragging its body along the floor of the pen over a time period of approximately 2 minutes (rising score 2). Click here for file

Additional file 2 **L-type BSE (L1) L-type BSE-inoculated steer at 17 mpi (prior to cull), 'nervous form'**. This steer displays head shyness in the crush (head shaking or tossing when observed from the front) and over-reactivity to testing of the menace response and touching of the head and upper neck with artery forceps. It refuses to walk towards the end of the corridor ('freezes') on its own but runs with hypermetric hind limb movements in this direction when other cattle are led out. It has considerable difficulty in rising (animal marked by arrow) with dragging its body along the floor of the pen (rising score 2). Click here for file

Additional file 3 **Control (CO1) Control steer (not inoculated) at 23 months after inoculation of test groups**. This steer mainly keeps its head towards the floor of the crush during cranial nerve assessments and there is only very mild over-reaction to testing of the menace response. It is willing to run along the corridor, only stopping briefly to inspect the door. It gets up without difficulty in the pen. Click here for file

Additional file 4 **H-type BSE (H4a) H-type BSE-inoculated steer at 18 and 20 mpi, 'nervous form'**. This steer startles and runs away after approaching and sniffing the camera at the door whereas the superimposed clip shows a control steer, observed in the pen for the first time at the same day, slightly backs off after sniffing the camera. Two months later it stands on its own in the other half of the pen and displays a spontaneous whole body flinch. Click here for file

Additional file 5 **H-type BSE (H4b) H-type BSE-inoculated steer at 21 mpi (prior to cull), 'dull form'**. This steer stood for considerable time in the pen with low head carriage whilst its pen mates were lying ruminating in the other half of the pen. It does not rise upon entering the pen when surrounded by its pen mates but rises when the rump is touched, dragging its body along the floor (rising score 2). Cranial nerve assessments are well tolerated. Its behaviour in the corridor is unremarkable (no signs of apprehension). It displays mild hind limb hypometria, best seen when the animal turns at the end of the corridor. Click here for file
